# Supplementary material for: ZC3H4 restricts non-coding transcription in human cells
Source: eLife. 2021 Apr 29;10:e67305. doi: 10.7554/eLife.67305 (PMC8137146; doi:10.7554/eLife.67305)

Figure 1 (B) – αCPSF30

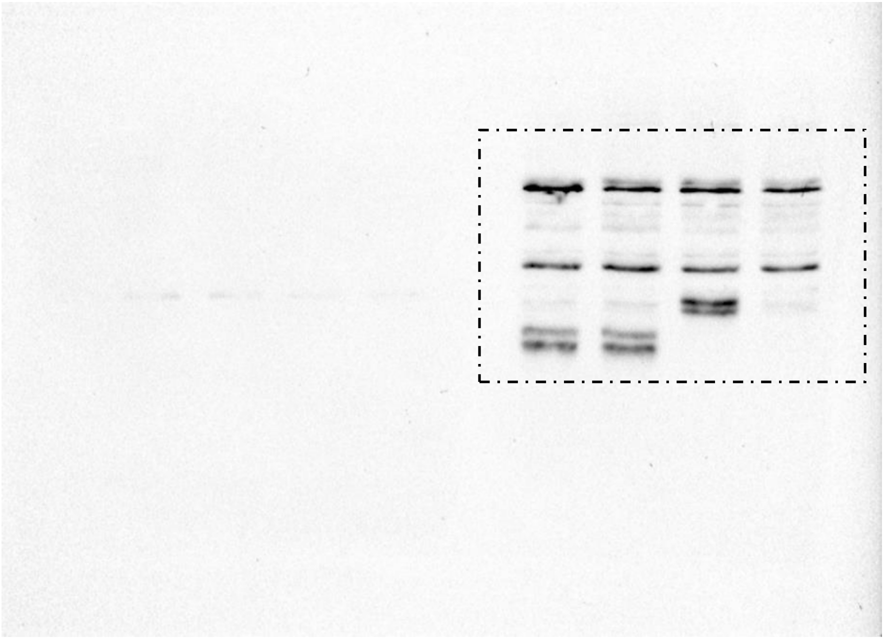

Figure 1 (E) – strep-HRP

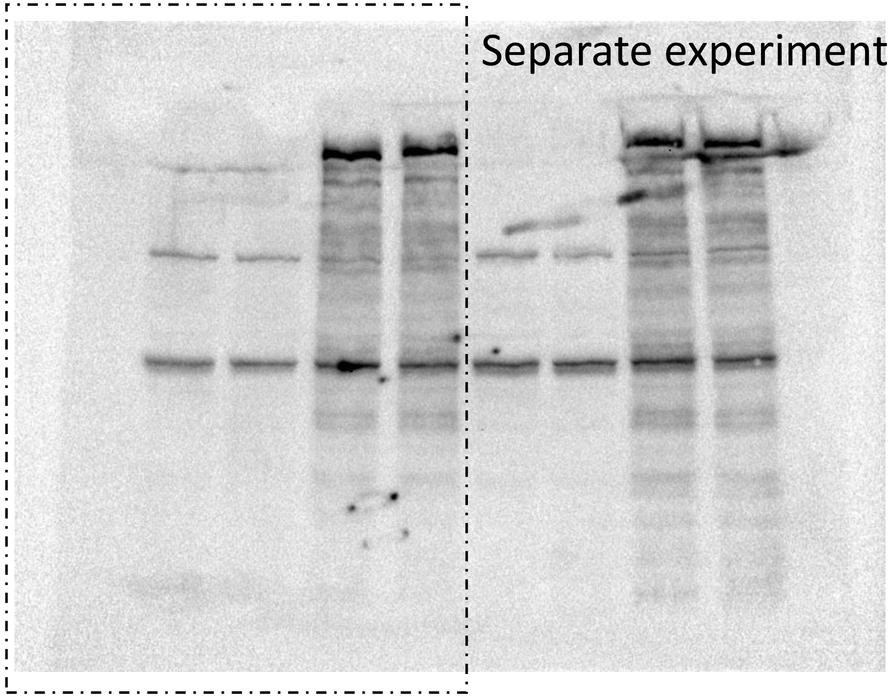

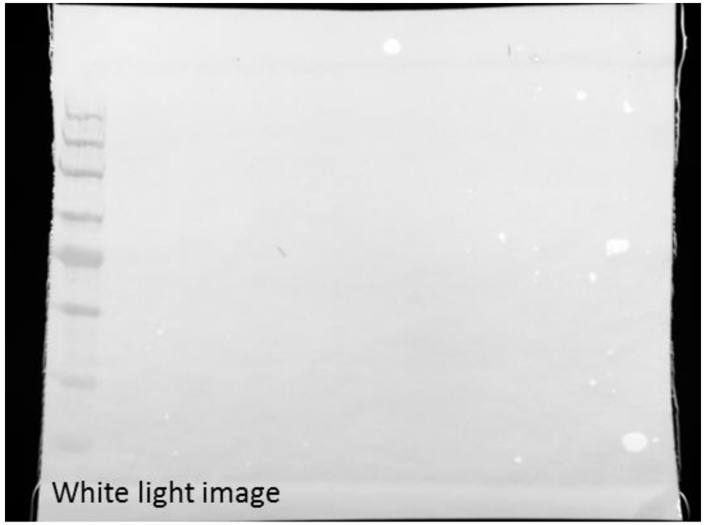

Figure 4(B) - αHA (Z4) / EXOSC10

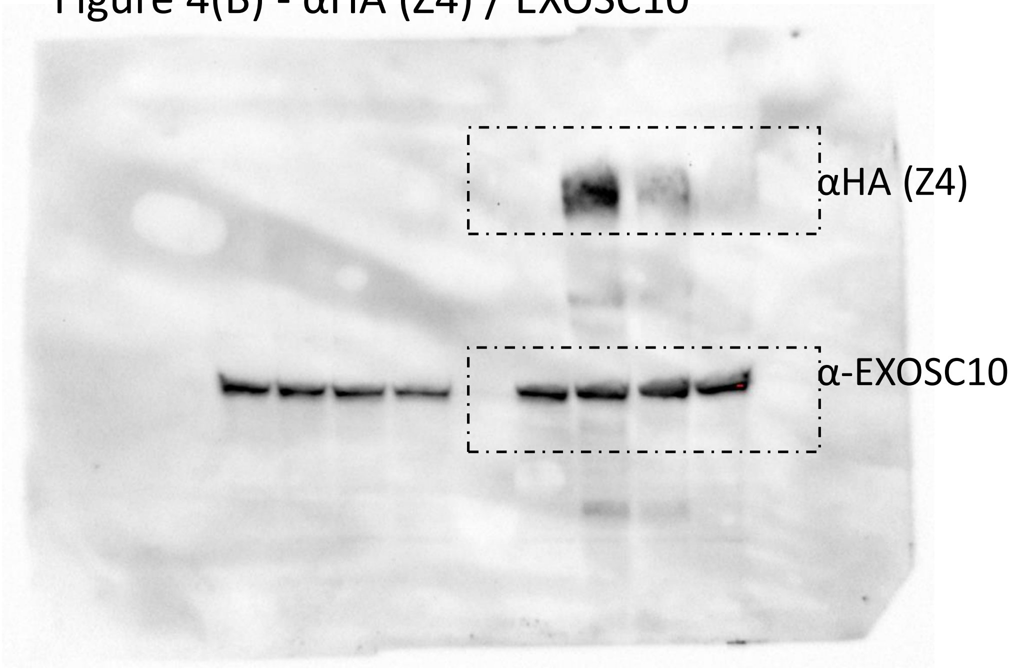

Figure 4 (B) – αZC3H4

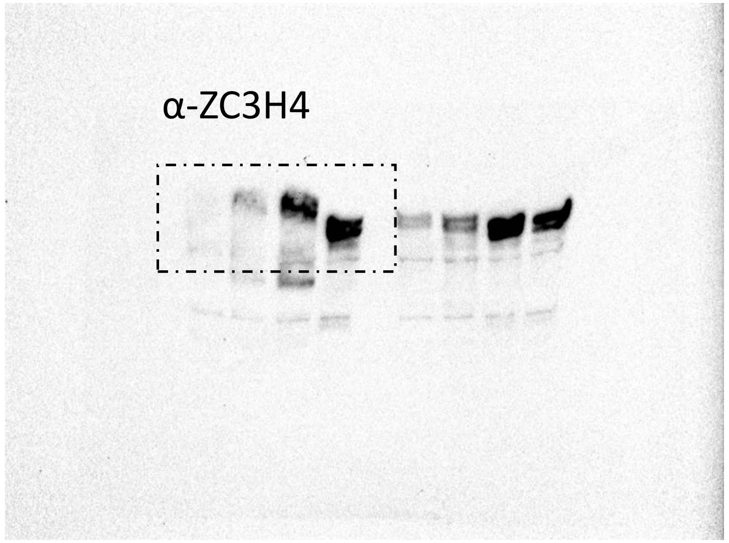

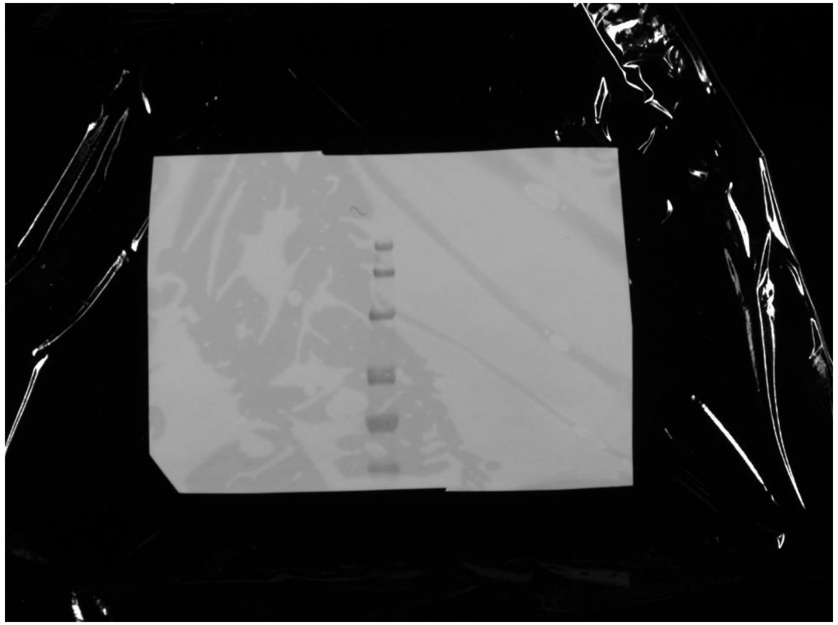

Figure 4(C) - αHA (Z4)

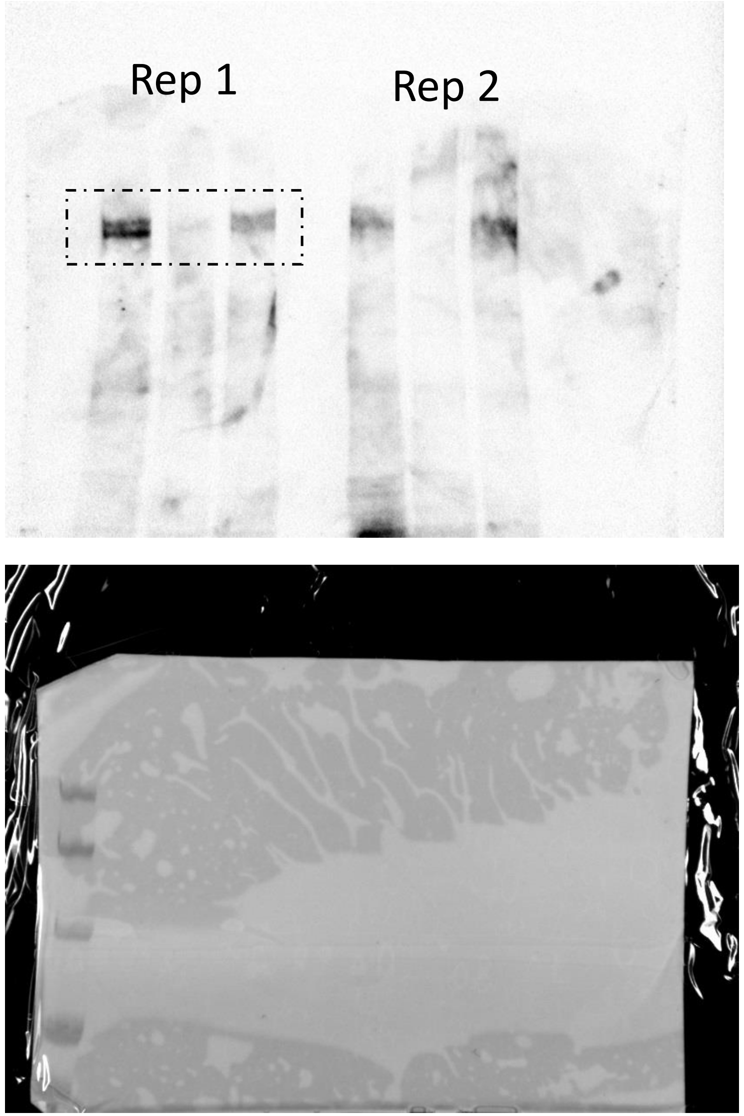

Figure 4(C) – αEXOSC10

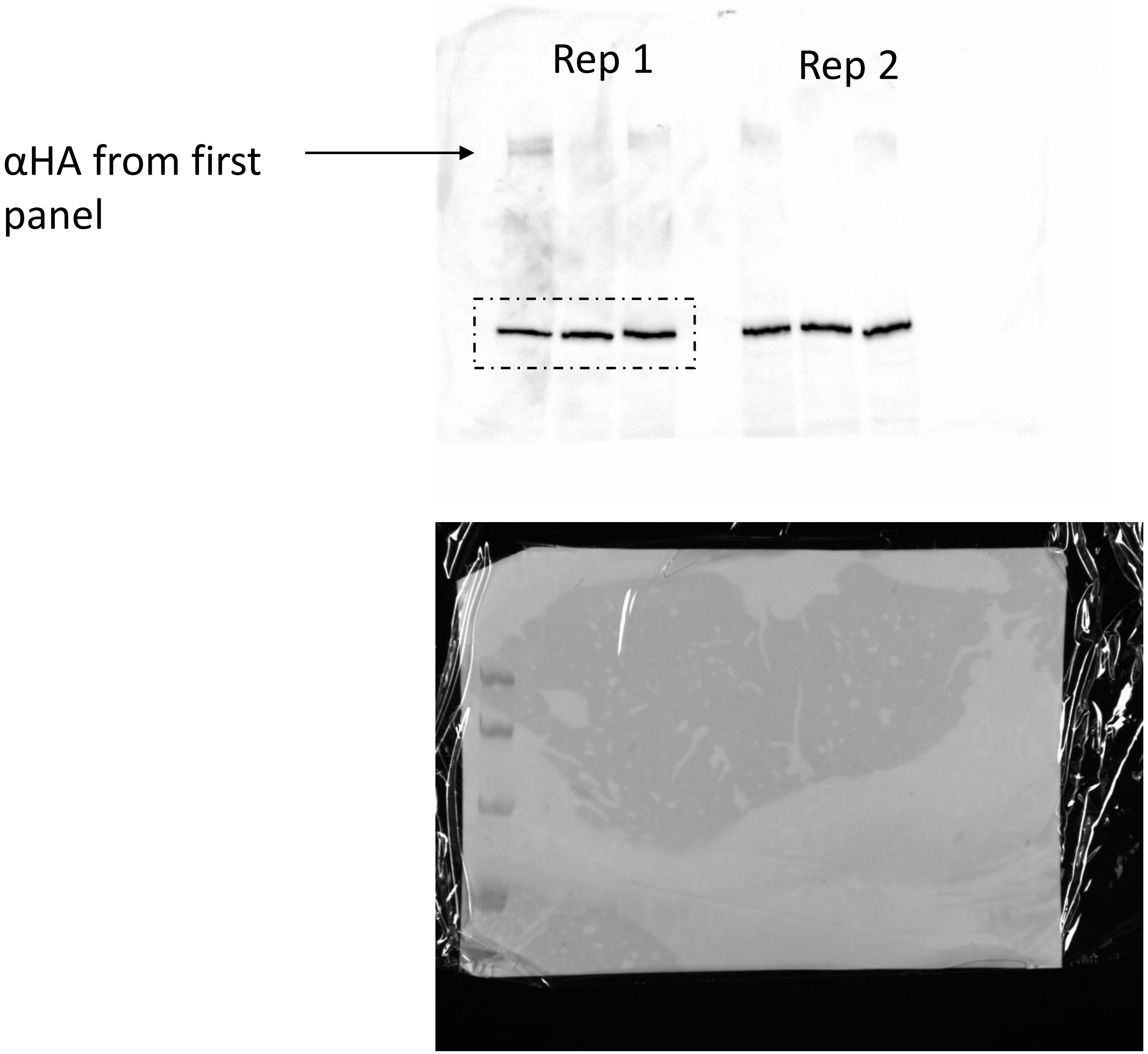

Figure 4 (F) – WDR82

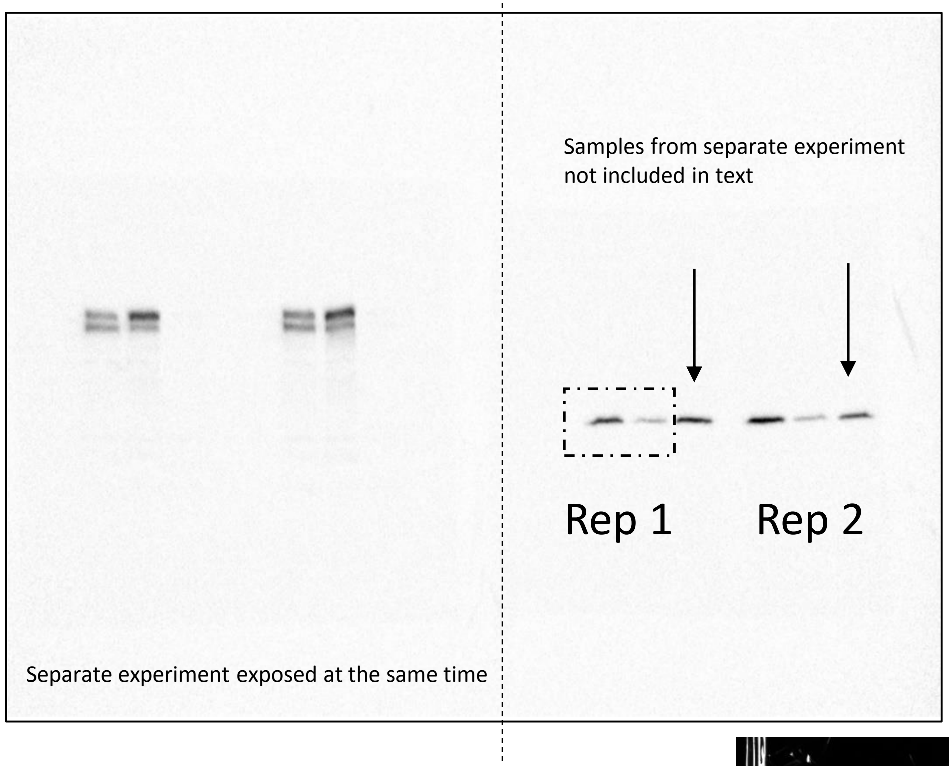

Figure 4 (F) – EXOSC10 (loading for WDR82)

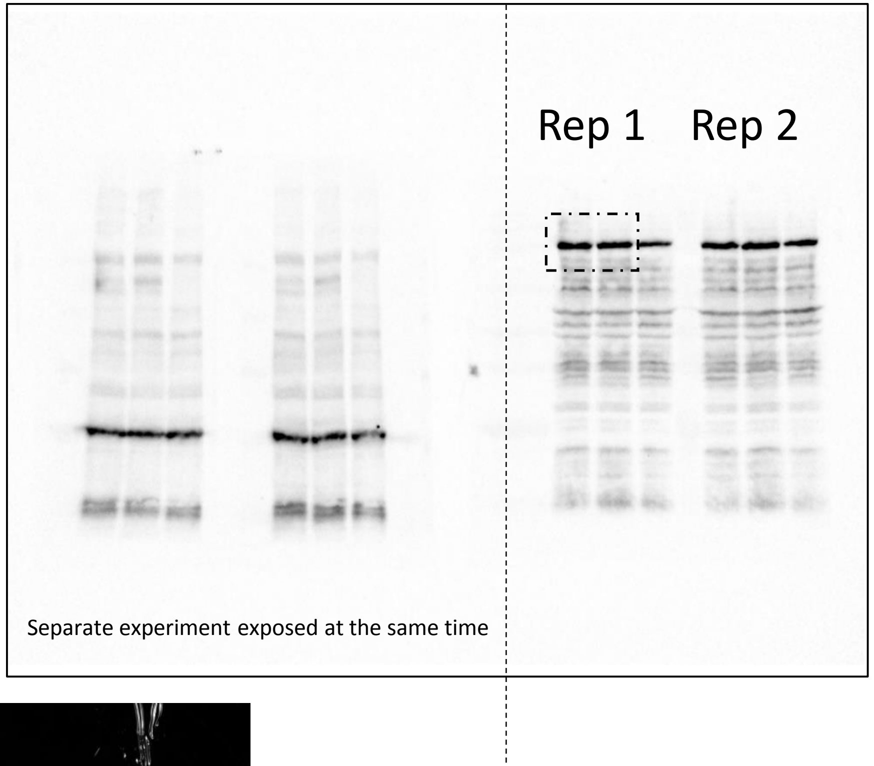

White image

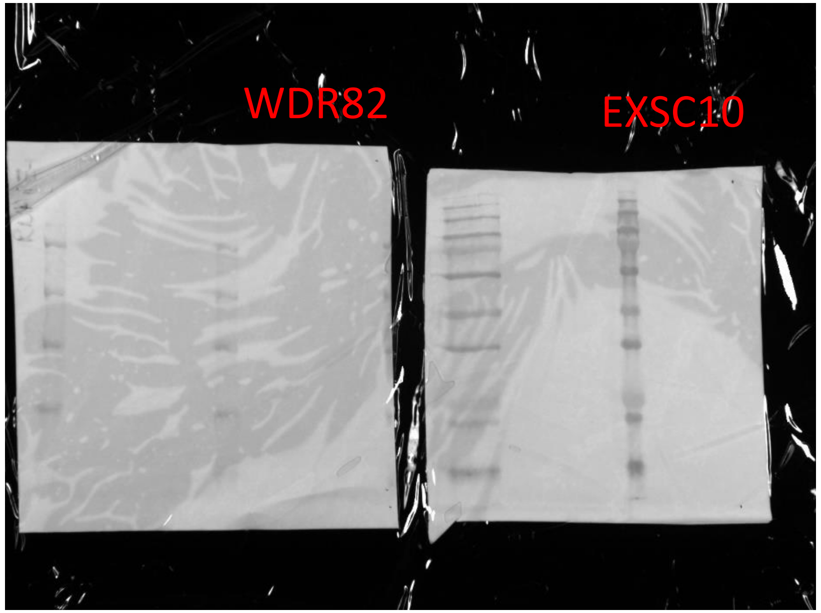

Fig 1 – fig supplement 1(B) – Pol2

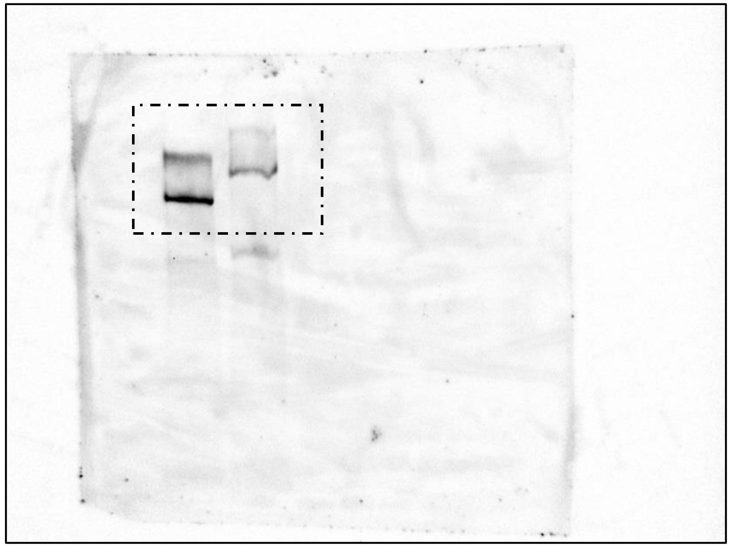

Fig 1 – fig supplement 1 (B) – EXOSC10 (loading)

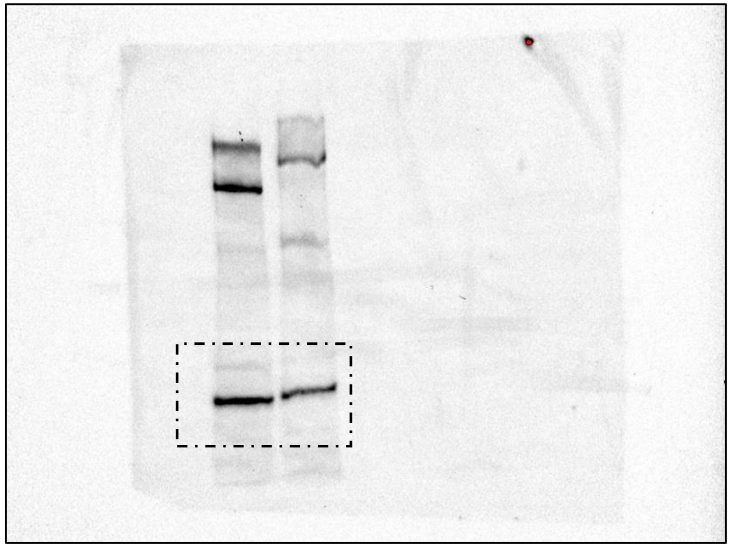

Fig 1 – fig supplement 1 (B) – white light image

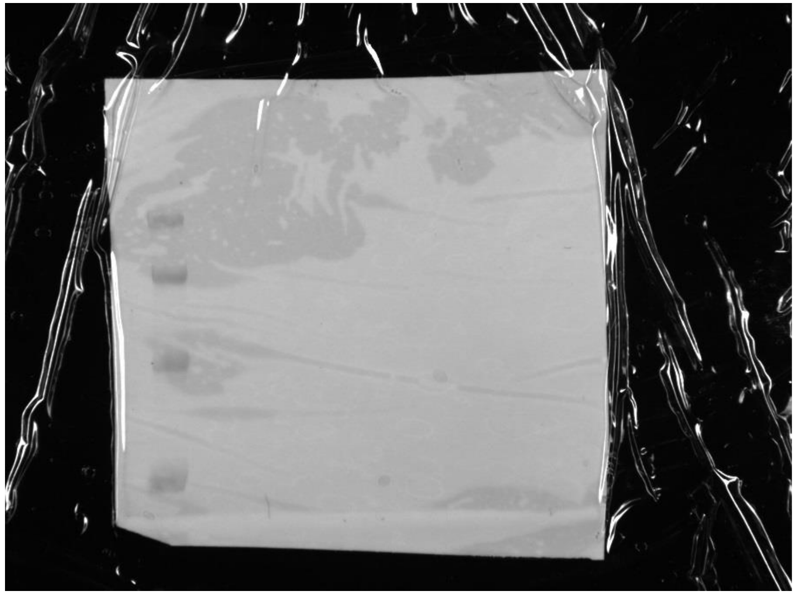

Fig 1 - fig supplement 2 (D) αGFP

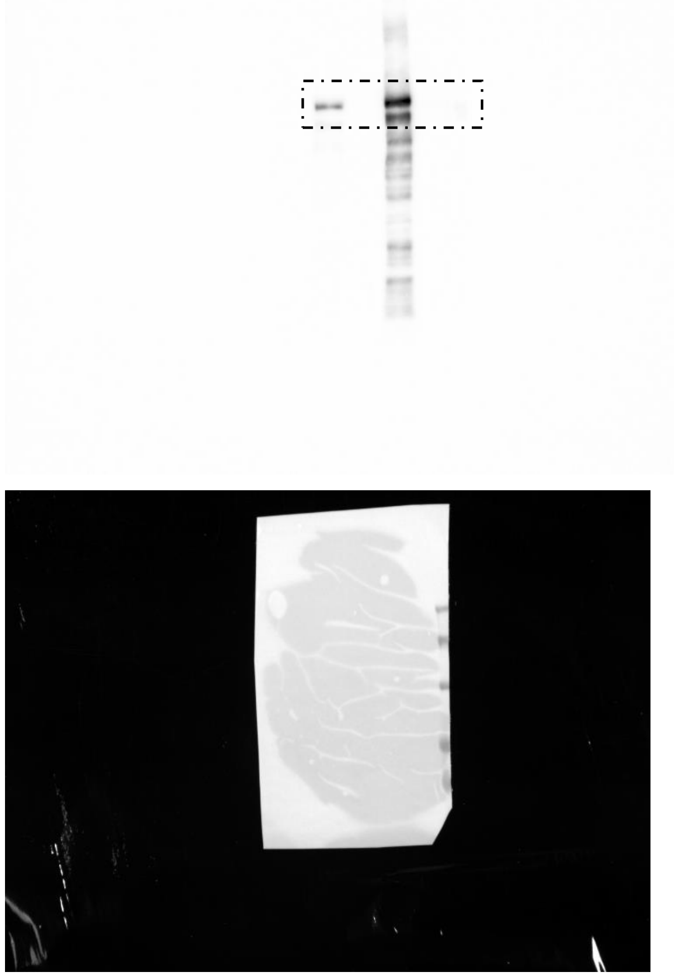

Fig 1 – fig supplement 2 (D) – αWDR82

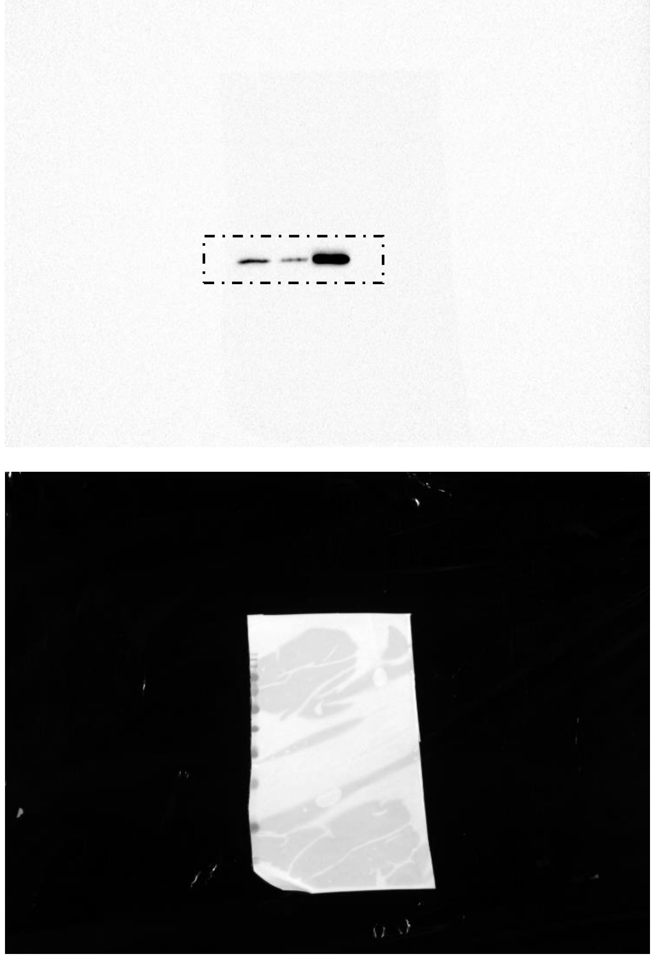

Fig 2 – fig supplement 1 (A)

αEXOCS10

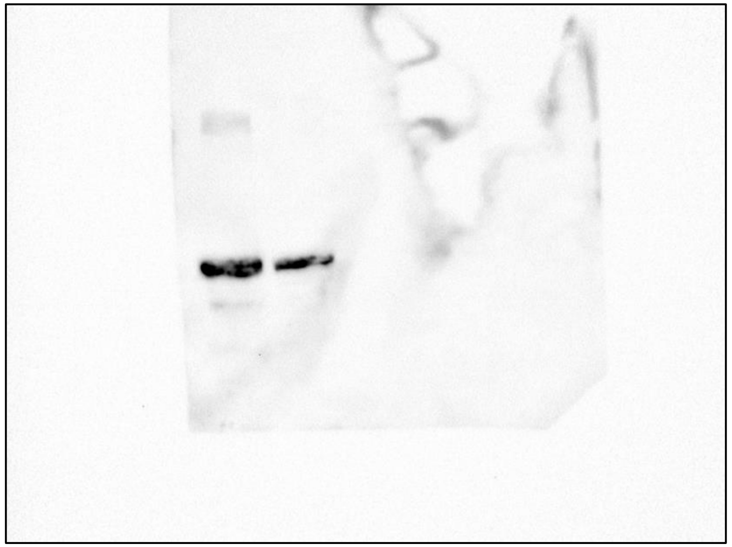

White

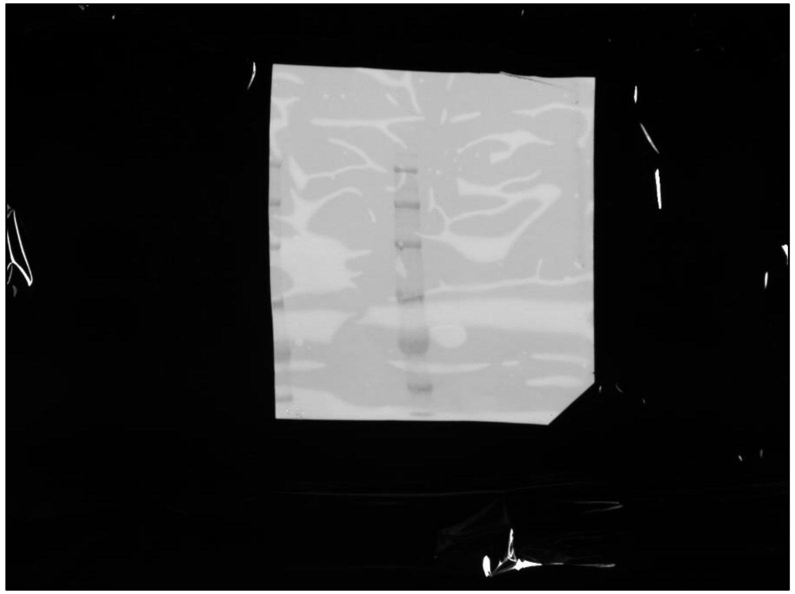

αZC3H4

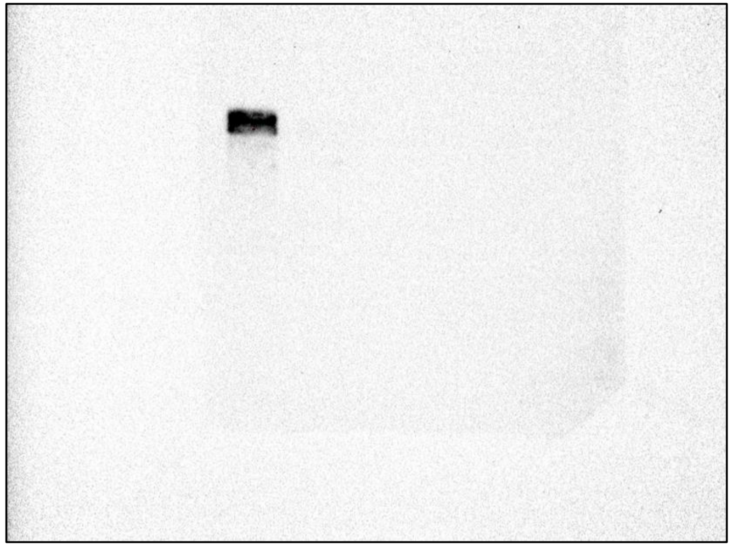

Fig 4- fig supplement 1 (C) - αPNUTS

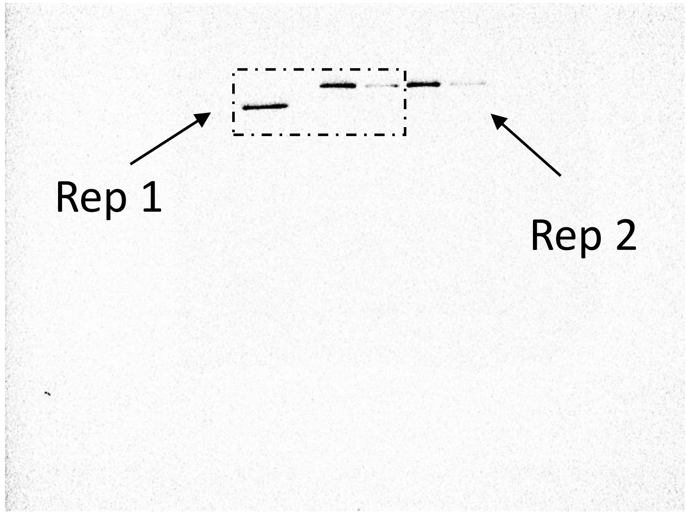

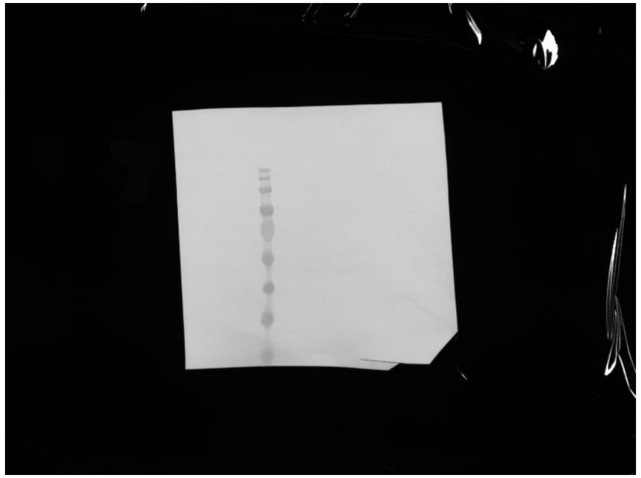

Fig 4 – fig supplement 1 (C) – αWDR82

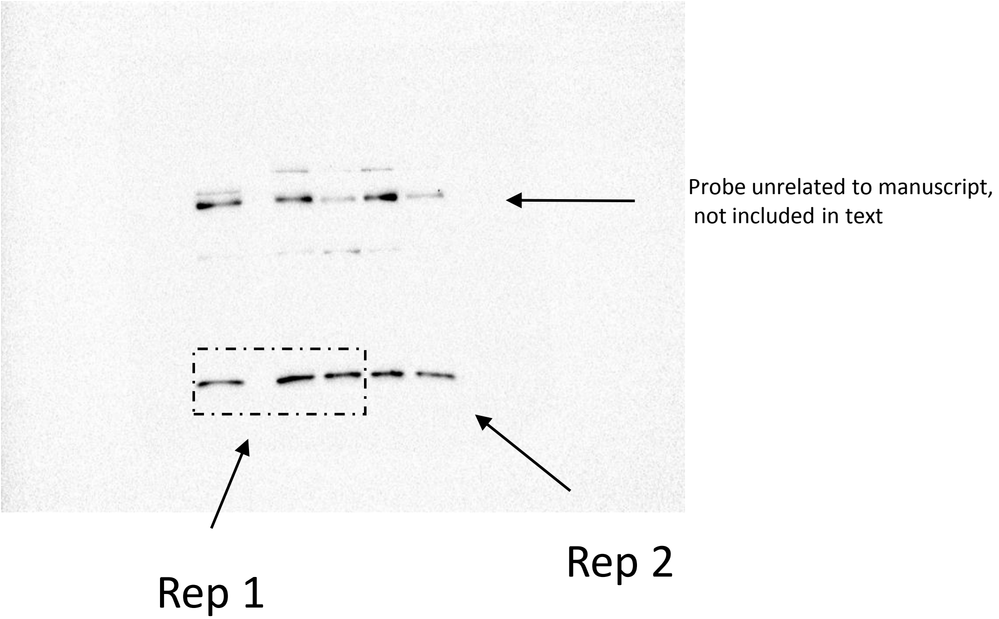

Fig 5-fig supplement 1 (A) – Upper panel

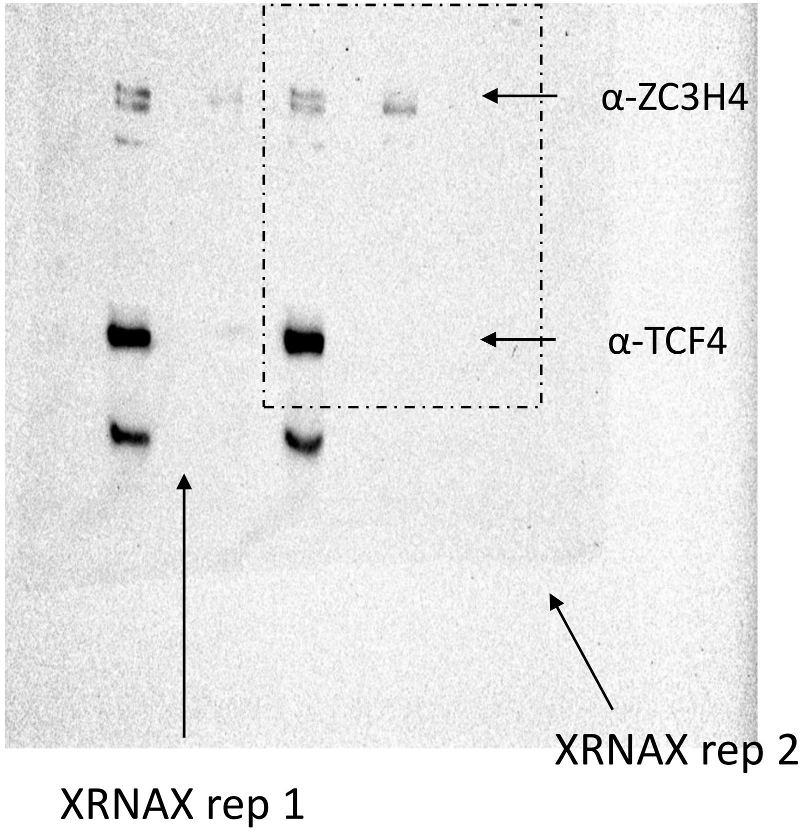

Fig5-fig supplement 1 (A)– EXOSC10

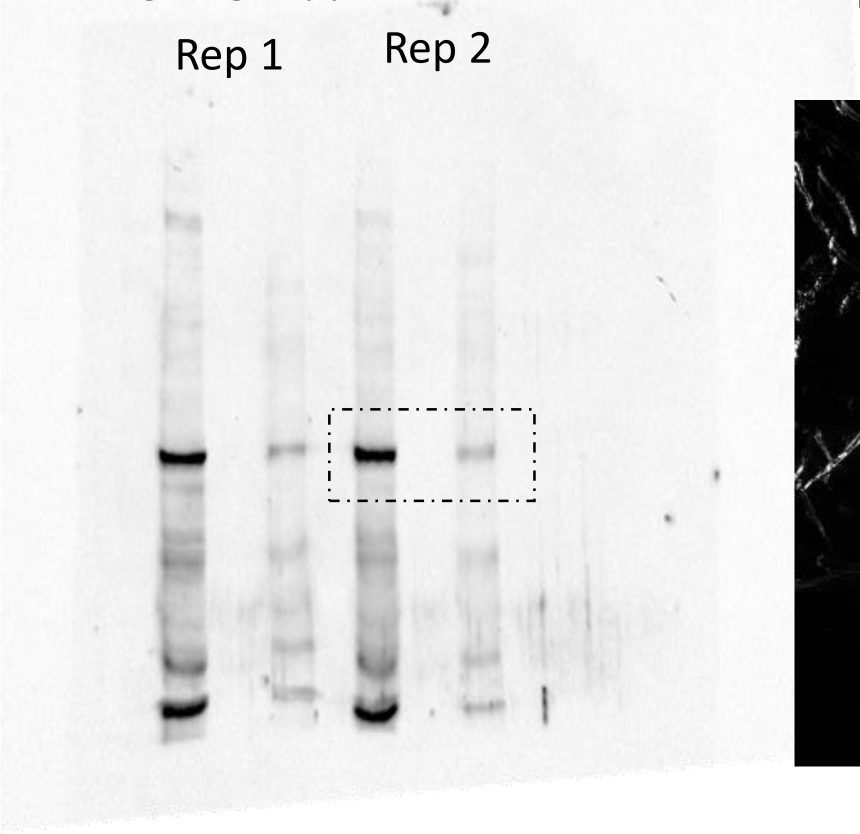

Fig 5-fig supplement 1(A)– White light image

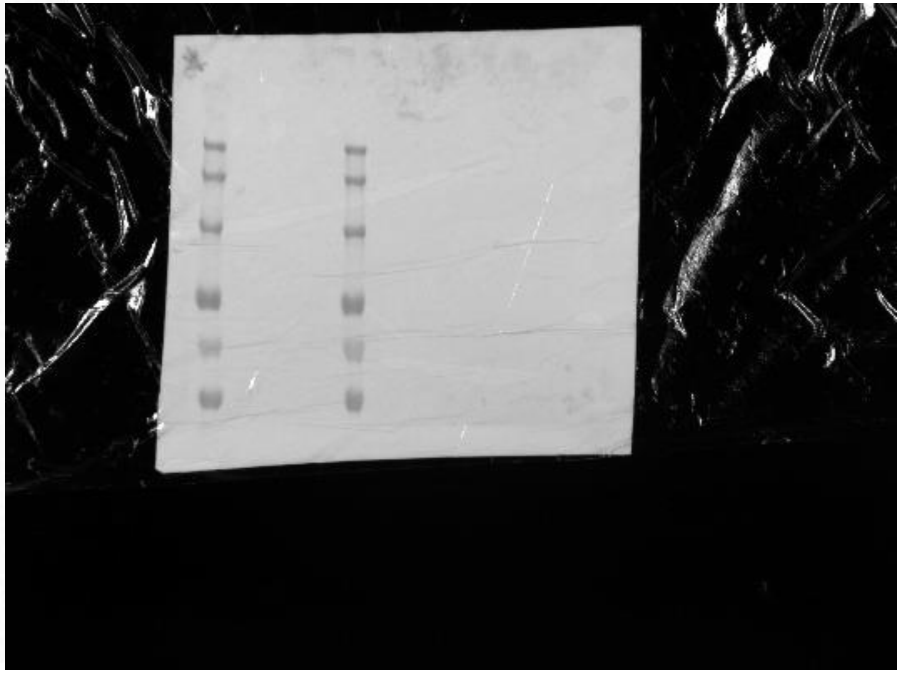

Supplement: Source data 2. [file elife-67305-data2.pdf]
